# Reliability of a New Bite Force Measure and Biomechanics of Modified Long Attack in Police Dogs

**DOI:** 10.3390/ani11030874

**Published:** 2021-03-18

**Authors:** Heli K. Hyytiäinen, Laura Blomvall, Mikko Hautala, Anu K. Lappalainen

**Affiliations:** 1 Department of Equine and Small Animal Medicine, Faculty of Veterinary Medicine, University of Helsinki, P.O. Box 57 (Viikintie 49), 00014 Helsinki, Finland; laura.blomvall@helsinki.fi (L.B.); anu.k.lappalainen@helsinki.fi (A.K.L.); 2 Department of Agricultural Sciences, Faculty of Agriculture and Forestry, University of Helsinki, P.O. Box 27, 00014 Helsinki, Finland; mikkoihautala@gmail.com

**Keywords:** working dog, police dog, long attack, functionality, clinical biomechanics, kinetics, kinematics

## Abstract

**Simple Summary:**

Information on the biomechanics of police dogs’ tasks is important in preventing, diagnosing and treating their work-related injuries and dysfunctions. Despite the fact that dogs in several fields of service are performing protective tasks, there is lack of information regarding the occupational physical demands these dogs are subjected to. This study aimed to develop and test a measurement sleeve for measuring dogs’ functional bite force, and to report locomotion-related values during a modified long attack. The measurement sleeve was found to be reliable, although individual tooth force could not be reported, as the forces were above the scale of our sensors. The dogs’ jaws pressing force, on the other hand, was not high, whereas during acceleration and deceleration the dogs were subjected to relatively high gravitational force equivalents. There are differences between two breeds of police dogs’ locomotion during the modified long attack task. The results gained from this study provide information which can be used to benefit the working dogs’ welfare during their use and training, as more in-depth understanding of the strains to their neuromusculoskeletal system is available.

**Abstract:**

Information on the biomechanics of police dogs’ tasks is important in understanding their work-related injuries and dysfunctions. This study aimed to develop and test a measurement tool for dogs’ functional bite force and to report modified long attack-related kinetic and kinematic values. Twenty Finnish male police dogs, 7 German Shepherd Dogs (GSDs) and 13 Belgian Shepherd Dogs, Malinois (BSDMs), were included. Dogs accelerated 25 m and bit the helper’s sleeve, fitted with three force sensors. Dogs were wearing a 3D accelerometer and were videotaped with a high-speed camera. The sleeve’s reliability for measuring the dog’s bite force was evaluated via intraclass correlation and Cronbach’s alpha. Otherwise, a Mann–Whitney U-test was used, with significance set at *p* = 0.05. The sleeve’s test-retest reliability was moderate to good (intraclass correlation of 0.75), and internal consistency was high (Cronbach’s alpha 0.75). The GSDs’ median bite force was 360.4 N (interquartile range (IQR) 628.6 N) and BSDMs’ 247.0 N (IQR 289.8 N). Median acceleration maximum was 7.1 gravitational force equivalent (g) and median deceleration maximum was 11.6 g, with highest recorded forces being 9.2 g and 13.1 g, respectively. The measurement sleeve was a reliable tool for measuring functional bite force in GSDs and BSDMs. Forces related to bite, approach and impact in the two breeds were reported.

## 1. Introduction

German Shepherd Dogs (GSDs) and Belgian Shepherd Dogs, Malinois (BSDMs) are traditionally the most commonly used dog breeds in police forces and in the army [1,2,3]. Police dogs’ duties and the schutzhund sport discipline (Internationale Gebrauchshund Pruefung, IGP) include similar physical elements and challenges [1,4]. The IGP as a sport comprises several different sections and tasks within these sections [4]. Long attack is one of the most commonly known tasks in the protection section of the IGP, and it is also part of police dogs’ work and training. Essentially, during the long attack the dog will accelerate towards the helper over a distance of 30–40 m, attack the bite sleeve and release the bite on command of the instructor.

Long attack can biomechanically be divided, from start to bite, into phases of acceleration, steady state running, deceleration and impact. Bite is the final result of the attack. Various mathematical methods of calculating dogs’ bite force (i.e., the pressing force of the dog’s jaws) post mortem have been published [5,6,7,8,9,10]. However, it has been suggested that these methods underestimate the force measured from a living dog [7]. Some of the studies on living dogs, in turn, have been undertaken by the method of anaesthetized dogs’ having electricity applied to their jaw musculature [7,11]. Functional force refers to the force produced during a task that the dog performs actively that corresponds to one they would perform as part of their daily life; work or sport. The results of the earlier studies cannot be regarded as functional, not only because forces are measured from the tips of certain teeth but also because the bite force has been passively induced, lacking conscious control based on teeth’s proprioceptive feedback, as well as the influence of the dog’s biting motivation. Whilst studies looking at various factors relating to dogs’ acceleration have been published [12,13,14], deceleration has been mostly disregarded; and neither of the two have been looked at in relation to police dog’s work.

The forces subjected to the working dogs’ musculoskeletal systems have not been reported to date. Moreover, to our knowledge, there are no publications on work-specific biomechanical factors, despite the obviously high level of physical demand in it. Measuring task-related kinetic and kinematic values would yield important knowledge of associated risks, providing information that can help when training dogs or in preventing and treating occupational injuries.

The aim of this study was to develop and test a tool for measuring protection dogs’ functional bite force. Acceleration and impact-related values are also reported. We hypothesized that the measurement sleeve developed as part of this study is reliable and produces repeatable results of dogs’ bite force.

## 2. Materials and Methods

The study protocol was approved by the University of Helsinki Ethics Review Board at Viikki Campus, Finland, and written consent was provided by dogs’ owner. The experiments were conducted in accordance with the animal welfare regulations and guidelines of Finland.

All Finnish police dogs in active duty (*N* = 240) were invited to participate in the study. Based on statistical analysis and previous similar publication [15], 20 dogs were estimated to be sufficient for testing the reliability of the measurement tool. Male police dog, either GSD or BSDM, in active duty was the inclusion criterion. Exclusion criteria were dogs not trained in use of force, dogs with any known mouth, tooth or musculoskeletal system-related injury or disease, and dogs with findings at the veterinarian’s on-site assessment.

The dogs were weighed and their height at withers measured on site. Their general health and ability to participate in the study were evaluated by a veterinarian, author Anu K. Lappalainen (AKL). All dogs were warmed up according to their own routines prior to performance, outside the testing arena, which was an indoor football arena with an astroturf surface. Dogs wore a standard police dog harness, fitted to each dog individually. All dogs were allowed to familiarize themselves with their surroundings—the indoor arena, present personnel, the equipment at site and worn by them—before the data collection, to ensure that no effect to dogs or their performance was seen due to these factors.

**Figure 1:**
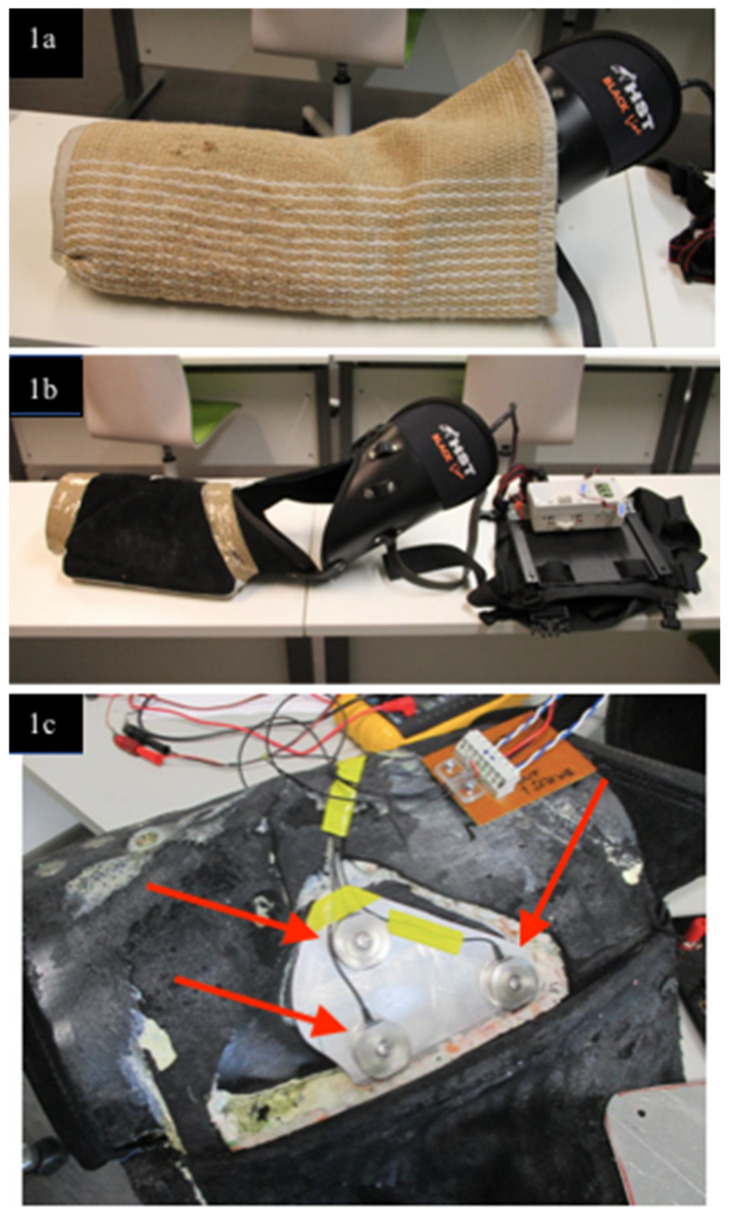
(**1a**) Left-hand bite sleeve with embedded measurement equipment. (**1b**) The bite sleeve without the surface material. (**1c**) Three force sensors (indicated by arrows) fitted into the deepest layer of the bite sleeves.

**Figure 2:**
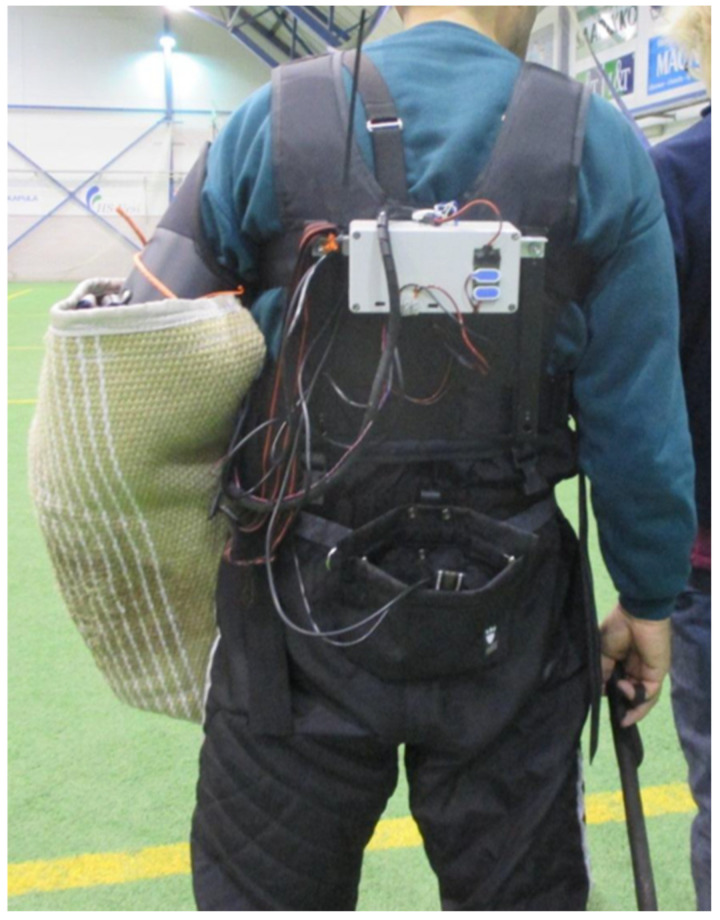
Measurement equipment on the helper.

The bite force was measured by customizing a regular helper sleeve with measurement equipment for the purposes of the study. A left-handed standard helper sleeve (HST Black Line, HST, Čechtín, Czech Republic) was fitted with three force sensors (FC23 Series Compression Force Sensors, Measurement Specialties Inc., TE Connectivity company, Fremont, CA, USA) (Figure 1) and rebuilt to maintain its original size, shape and bite feel. The professional helper using the sleeve and a veterinarian (AKL) confirmed that the modified helper sleeve resembled the original and that it was safe for the dogs. The sensitivity of the sensors was 20 mV/V, input voltage 9 V and the full range of each sensor 113.4 kg = 1112.1 Newtons (N). The sensors were in parallel, and thus, the output was 20/3 × 9 mV = 60 mV for full range. The signal was amplified by a factor of 100 and the data then collected using a LabQuest 2 data collector (Vernier Software and Technology, Beaverton, OR, USA) using a voltage probe VP-BTA (−10 V–+10 V). The maximum force that could be measured was thus 10 V/6 V×50 lbf = 420 lbf = 2000 N, corresponding to 200 kg. The sensors aimed to measure the biting force of the jaws of the dog. Above these, a folio type of membrane was set (L-series sensor, Emfit Ltd. Sensor Products Division, Vaajakoski, Finland) to measure the dynamic voltage and its changes, i.e., the pressing force of each individual tooth. The membrane was covered with a 3 mm-thick rubber mat and suede leather (original sleeve materials). The equipment on the helper is presented in Figure 2. The aforementioned raw data were analysed using LoggerLite 1.7 software (Vernier Software and Technology).

The helper was a professional police dog trainer, familiar to all of the dogs. All the dogs were allowed to bite the sleeve once at the starting place before the actual test. The helper instructed and approached each dog in a standardized manner. Dogs were handled by their own police officer or trainer. There were no other dogs in the vicinity during the testing. The long attack normally includes acceleration, steady state running and deceleration over 30–40 m. We modified the task by decreasing the distance to 25 m due to space limitation. The dogs were placed on a site on a line 25 m away from the helper. On command, the dogs accelerated and attacked the helper, biting the sleeve. As the attack was completed, the helper instructed the dog’s handler to give the release command, after which the handler retrieved the dog and returned it to the starting position. Each dog repeated the modified long attack three times.

All dogs’ performances were videotaped with a two-dimensional high-speed video (Casio EX-FH20, Casio Computer Co., Ltd., Tokyo, Japan) with 224 × 168 recorded pixels and 420 frames per second. The camera was set laterally from the line where the dog met the helper, and vertically at the level of the helper’s arm and sleeve, 8 m away from the meeting point of the dog and the helper, which was marked on the ground. The height of the sleeve was kept at approximately 90 cm. The height of the helper was 181 cm, which on the screen was 9.8 cm. This height was used as a reference on the screen, by which the jump distance for the dogs was measured by using a coefficient of 18.5, based on the helper’s height (181 cm/9.8 cm). Measurements were taken in millimeters on screen from the cranial border of the toes of the limb last in contact with the ground before the bite, to the toes of the helper. The computer screen was 33.8 cm in diameter, with resolution of 1440 × 900.

**Figure 3:**
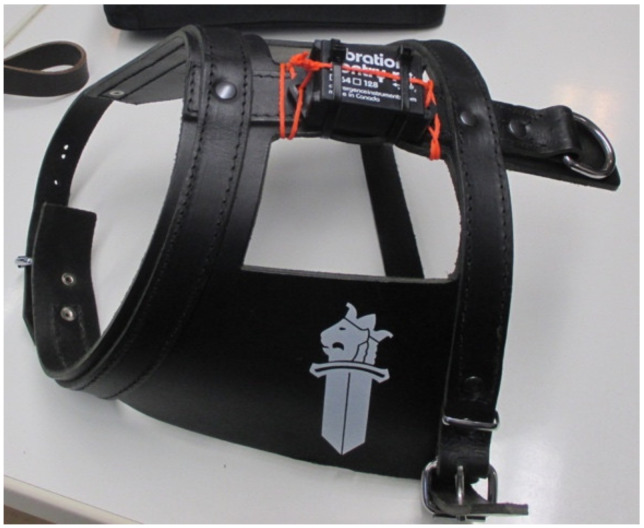
Three-dimensional accelerometer attached to dogs’ harnesses.

A three-dimensional (3D) accelerometer was attached to a fitted harness during all of the repetitions of the modified long attack (Figure 3), to collect acceleration and deceleration data (gravitational force equivalent, g) during the performance. Similar placement site for the accelerometer was used, as in another recently published experiment [16]. The 3D acceleration was measured with a Vibration Sentry RT 64 g data logger (Convergence Instruments, Sherbrooke, QC, Canada) using a recording rate of 100 Hz, saving the mean, maximum and minimum acceleration of each axis in 5 s epochs. In addition, a standard police radar was used to measure the speeds of the dogs during acceleration.

A sample of 20 dogs was estimated to be sufficient for the reliability testing of the study, but for the breed comparison-related methods, the unequal groups were small in number. Due to the small sample size, non-parametric statistical methods were used to analyse the data. For descriptive statistics, medians of pooled repeated measures were used. Using the SPSS statistical software (Version 25, Microsoft, Armonk, NY, USA), Mann-Whitney U-test was used to analyze the age, weight, height and bite force differences between the two breeds. The same method was used to analyse the acceleration and deceleration values. Reliability of the measurement sleeve was evaluated via Cronbach’s alpha and intraclass correlation. Regarding Cronbach’s alpha, 0.70–0.95 is considered an acceptable value for any test used in clinical human medicine [17]. The test-retest reliability, when tested via intraclass correlation, is classified as follows: <0.5 = poor, 0.5–0.75 = moderate, 0.75–0.9 = good, >0.90 = excellent [18]. Level of statistical significance for all analyses was set at *p* = 0.05. All results are reported as medians due to the use of non-parametric tests and the small sample size.

## 3. Results

Twenty Finnish male police dogs, 7 GSDs and 13 BSDMs, participated in the study. Different number of these dogs were used for the sleeve-related study section, and for the acceleration-related study section, due to data corruption in either section for some dogs. The descriptive information on the dogs used for these two study sections, and the two breed groups within these groups, is presented in Table 1. Weight was the only statistically different conformational factor measured between the groups: the GSDs were significantly heavier (*p* = 0.001 for both groups) than the BSDMs.

The median (interquartile range (IQR)) bite force of all dogs was 269.7 (392.9) N. No significant difference emerged in the median bite force, i.e., the pressing force of the jaw, between the GSDs and the BSDMs (*p* = 0.151). The difference in median maximum force between the breeds was 113.4 N (Table 2). The test-retest reliability of the sleeve was found to be moderate to good, with an intraclass correlation of 0.75. Cronbach’s alpha was 0.75, suggesting relatively high internal consistency for the measurement sleeve.

The dynamic voltage, i.e., the pressure of each individual tooth, could not be reported due to it being higher than the expected values, exceeding the equipment’s measurement capacity. One GSD and one BSDM had to be excluded from the sleeve and bite force-related data analysis due to raw data corruption.

Acceleration (7.1 g) or deceleration (11.6 g) values of the two breeds did not show statistically significant differences (*p* = 0.438 and *p* = 0.311, respectively) (Table 2). The radar measurements were not successful in many of the dogs due to the dog being a small target in a large space; only 3 GSDs and 10 BSDMs had a recorded value. The speeds ranged between 35 km/h and 42 km/h (median 37 km/h, IQR 0 km/h) for GSDs, and between 29 km/h and 41 km/h (median 40 km/h, IQR 0 km/h) for BSDMs. The range of speeds was from 29 km/h to 42 km/h for all 13 dogs (median 40 km/h, IQR 0 km/h). 

The median distance of jump to the sleeve for all dogs was 1.2 m (IQR 0.7 m), and no significant difference was present between the two breeds in the length of jump (*p* = 0.064) (Table 2). One BSDM had to be excluded from the jump distance measurements due to poor video quality, and one GSD and one BSDM had to be excluded from the jump distance-related data analysis due to raw data corruption.

## 4. Discussion

Based on our results, the measurement sleeve developed for this study is a reliable tool for measuring protection dogs’ functional bite force. An important difference in our study relative to previous ones is the different approach to the bite. Our aim was to measure the functional bite force in a real-life situation, not just bite force. Studies on dogs’ functional bite force are limited, and the only one to our knowledge is a paper by Lindner et al. (1995), which presented the mean values for 22 different breeds of dogs that bit a steel rod coated with a tasty leather. The mean bite force of the whole group was 256 N, varying between 13 N and 1394 N. Two of the dogs were Belgian Shepherd Dogs, Tervueren, and their bite force was reported to be 137 N and 367 N. These values resemble the ones we recorded for the BSDMs (median 246.8 N). However, Lindner’s results are not applicable to police or IGP dogs. These dogs are trained protection dogs of certain breeds, with high motivation levels, which bite the sleeve in conjunction with impact after a high speed. Moreover, the rod used in Lindner’s study was not validated, nor was it studied for its reliability. Thus, our sleeve seems to be the first reliability-tested method for measuring these forces functionally.

Bite force increases as the length of the dog’s muzzle decreases and the size of the dog or its head increases [8]. In our study, the difference in the anatomical structures of the head and temporomandibular joint between the two breeds, GSD and BSDM, was not measured. Thus, the difference in bite force cannot be concluded based on this study. Although the difference in bite forces between the breeds was clinically obvious: 113.4 N, which corresponds to 31.5% of the GSDs’ and 45.9% of the BSDMs’ median force, it was not statistically significant. The population regarding this part of our study was limited, leading to a large variation in GSDs’ results and lack of a statistical difference between the breeds. Thus, further studies to confirm our preliminary results regarding the normal values of the two breeds are warranted.

To make our bite force-related findings more concrete, a comparison with human hand grip force can be made. Albeit the measurement tools and methods between the human hand grip studies and our study are not directly comparable, as the accuracy between the measurement tools is not validated, the comparison of the actual amount of reported forces is descriptive of the amount of functional forces involved in biting. The bite force of the GSD jaws reported in our study corresponds to the average grip force of a 70- to 79-year-old man’s hand. The BSDMs’ bite force, in turn, corresponds to the mean grip force of the hand of a man aged over 80 years [19]. This comparison clarifies the fact that the jaws of the dog have limits to their force production ability, and they also are limited by the structure and architecture of the temporomandibular joint and related structures; the pure biting force of the jaws is actually not that large. The key to the grip and damage caused by a dog’s bite are the teeth, but our study failed to record the tooth-by-tooth forces, as they were so high that they exceeded our equipment’s capacity.

In a relatively short distance, 25 m, these dogs reached speeds of up to 42 km/h. The dog’s acceleration towards the helper resulted in relatively high g-forces. With factors related to rapid acceleration combined with high speed [16], there is a risk of tissue damage already at the acceleration phase of the long attack. Again, to give a comparison with the human world, the acceleration peaks recorded in our study (median 7.1 g) are markedly higher than those experienced by formula one drivers during their races [20]. If continued over extended periods of time, this level of acceleration forces would be destructive to tissues; acceleration forces of 6–8 g over a period of 2–3 min have been shown to increase dogs’ relative pulmonary vascular resistance [21]. An even more noteworthy finding from our study was the deceleration values. The impact of the dog on the helper’s sleeve exposed the dog’s tissues to forces as high as 13 g. Compared with the acceleration over 25 m, the deceleration when hitting the sleeve and the helper is distributed over a relatively short period of time, thus making it more of a risk to the dog.

Our results did not show a statistical difference between the breeds in jump distance in their final approach. However, the fact that the BSDMs would jump to the sleeve at a median of 1.4 m away, whereas the GSD would jump 0.43 m (69%) closer to the helper suggests clinical importance, i.e., the one meter of difference in jump distance may, in practice, have implications to the dogs’ musculoskeletal system and performance. The lack of statistical significance surely arises from one of the biggest limitations of our study: the small final number of dogs and the unequal breed groups. Nevertheless, the difference between breeds’ performance techniques should be considered when training these two common police and IGP dog breeds. Moreover, the qualitative aspect of the approach should also be taken into account. While beyond the scope of our study, observations were made regarding the body positions of the two breeds during the approach, and further studies with larger sample sizes are warranted to investigate this aspect.

Several factors may have affected our results. The bite sleeve was a standard size sleeve, the same size for all dogs, as it is in normal life. The size and shape of a dog’s maxilla and mandible may affect the “grip” on the sleeve. One dog may open its mouth wider, thus gripping a larger portion of the sleeve, whereas another dog has a shorter mandible and is unable to bite with a “deep” grip. Based on basic physics, the closer the bite is to the joint’s axis, the larger the force. The leverage of the temporomandibular joint and the point of rotation in relation to the maxilla and mandible vary. This may affect the bite force of the dog. A recently published study reports, that especially mandible shape could be used as a predictor of bite force [22]. It has also been shown that the forces vary between the biting and non-biting side muscles, depending on the position during the bite [7]. The angle of our bite sleeve is approximately 50°. If a dog’s mouth is small, it must open the mouth wider, likely resulting in a weaker bite force [23]. Yet another related factor is that the dogs would not hit the helper and the sleeve identically, but would grab the sleeve at various angles and various points in relation to the sensors. This may have affected the bite force results.

Measuring functional performance of a dog may be affected by the personality and mental state of the dogs. The dogs in our study group were of different experience levels in this type of exercise. Despite using a familiar and standardized helper, some dogs might still become more excited than others, impacting the results.

Albeit our inclusion criteria stated that only dogs with no known musculoskeletal or mouth related issues would be included, and the dogs were assessed by a veterinarian, they were not radiologically examined at the time. Thus, it is possible, that the dogs may have had, for example osteoarthritis in their temporomandibular joints, it being the most common disease related to the temporomandibular joint in dogs [24]. Osteoarthritis might cause not only mechanical, but also pain-related limitations to the bite force [25].

In addition, the astroturf surface of the data collection site may have caused some dogs to be more vary in their speed, acceleration and/or deceleration. Although astroturf has not yet been studied for its effect on the dogs’ performance, relatively large amounts of displacement of paws during running has been reported on grass and turf [26].

It is important to note that the study failed to establish the pressure of individual teeth. All dogs bit in excess of our measurement tool’s available range (<300 N/cm^2^), clearly exceeding the maximum. Despite the lack of results in this regard, this finding has great clinical importance. People training these dogs should pay special attention to the health of the mouth, teeth and the temporomandibular joint, as the pressure they can withstand is key in bite performance, rather than the pressure force that the jaws can produce. This aspect of functional bite force should be investigated further.

## 5. Conclusions

In conclusion, a bite sleeve for measuring functional bite force was developed and found to be partially reliable. The functional bite force of two commonly used police dog breeds was measured to be lower than generally assumed, whereas the acceleration and deceleration values during the long attack were high. This should be taken into account when training dogs to long-attack types of task, as tooth, mouth, temporomandibular and global musculoskeletal health may be at risk due to these forces.

## Figures and Tables

**Table 1:** Descriptive information on the dogs included in all study sections.

Dog Groups	Number of Dogs	Median Age in Months (IQR)	Median Weight in kg (IQR)	Median Height in cm (IQR)
	Sleeve-related study sections			
All dogs	18	53.0 (43)	31.8 (7.8)	63.0 (2.3)
German Shepherd Dogs	6	58.5 (11)	35.2 (5.2)	63.0 (2.5)
Belgian Shepherd Dogs, Malinois	12	35.0 (54)	30.2 (5.4)	63.0 (2.8)
Difference between the two breeds		*p* = 0.250	*p* = 0.001 *	*p* = 0.964
	Acceleration-related study sections			
All dogs	20	56.5 (45)	32.5 (7.3)	63.0 (2.0)
German Shepherd Dogs	7	60.0 (11)	35.4 (8.0)	63.0 (2.0)
Belgian Shepherd Dogs, Malinois	13	41.0 (57)	30.6 (6.3)	63.0 (2.5)
Difference between the two breeds		*p* = 0.351	*p* = 0.001 *	*p* = 0.938

IQR = interquartile range; * = statistical significance.

**Table 2:** Descriptive information on bite forces, jump distance and maximal acceleration and deceleration forces of the dogs in this study.

Dog Groups	Number of Dogs	Median Bite Force (IQR)	Median Jump Distance (IQR)	Number of Dogs	Median Maximal Acceleration (IQR)	Median Maximal Deceleration (IQR)
All dogs	17	269.7 (392.9) N	1.2 (0.7) m	20	7.1 (2.0) g	11.6 (2.5) g
German Shepherd Dogs	6	360.4 (628.6) N	1.0 (0.4) m	7	7.1 (2.0) g	11.6 (2.5) g
Belgian Shepherd Dogs Malinois	12	247.0 (289.8) N	1.4 (1.0) m	13	7.1 (2.2) g	11.6 (2.2) g

*N* = Newtons; g = gravitational force equivalent; IQR = interquartile range, m = meters.

## Data Availability

Data is not available due to ethical and privacy limitations, based on the consent provided by the participants.
